# Cost avoidance of pharmacist-led deprescribing using STOPPFrail for older adults in nursing homes

**DOI:** 10.1007/s11096-024-01749-3

**Published:** 2024-07-05

**Authors:** Eoin Hurley, Stephen Byrne, Elaine Walsh, Tony Foley, Noel Woods, Kieran Dalton

**Affiliations:** 1 Pharmaceutical Care Research Group, University College Cork, Cork, Ireland; 2 Department of General Practice, University College Cork, Cork, Ireland; 3 Centre for Policy Studies, University College Cork, Cork, Ireland

**Keywords:** Frailty, Medication review, Nursing home, Pharmacist, Pharmacoeconomic, STOPPFrail

## Abstract

**Background:**

The Screening Tool of Older Persons Prescriptions in Frail adults with limited life expectancy (STOPPFrail) criteria aim to reduce inappropriate/unnecessary medications in frail older adults, which should minimise adverse drug events and additional healthcare expenditure. Little is known about the economic outcomes of applying these criteria as an intervention.

**Aim:**

To evaluate cost avoidance of pharmacist-led application of STOPPFrail to frail older nursing home residents with limited life expectancy.

**Method:**

Pharmacist-identified STOPPFrail-defined potentially inappropriate medications that were deprescribed by patients’ general practitioners were assigned a rating by a multidisciplinary panel, i.e. the probability of an adverse drug event occurring if the medication was not deprescribed. The intervention’s net cost benefit and cost–benefit ratio were then determined by factoring in adverse drug event cost avoidance (calculated from probability of adverse drug event ratings), direct cost savings (deprescribed medication costs/reimbursement fees), and healthcare professionals’ salaries.

**Results:**

Of the 176 potentially inappropriate medications deprescribed across 69 patients, 65 (36.9%) were rated as having a medium or high probability of an adverse drug event occurring if not deprescribed. With €27,162 for direct cost savings, €61,336 for adverse drug event cost avoidance, and €2,589 for healthcare professionals' salary costs, there was a net cost benefit of €85,909 overall. The cost–benefit ratio was 33.2 and remained positive in all scenarios in sensitivity analyses.

**Conclusion:**

Pharmacist-led application of STOPPFrail to frail older nursing home residents is associated with significant cost avoidance. Wider implementation of pharmacist interventions in frail older nursing home residents should be considered to reduce potentially inappropriate medications and patient harm, alongside substantial cost savings for healthcare systems.

**Supplementary Information:**

The online version contains supplementary material available at 10.1007/s11096-024-01749-3.

## Impact statements

- Application of STOPPFrail by a pharmacist to frail older adults in nursing homes was associated with significant cost savings due to prevented adverse drug events and deprescribed medications.
- The cost–benefit ratio for this pharmacist-led intervention remains positive in all scenarios when lower and upper limit estimates for costs and benefits were tested in sensitivity analyses.
- If no adverse drug events occurred during the 12 months following the intervention, the direct cost savings arising from deprescribed medications alone would cover the cost of the intervention at least five-fold.
- Medications without a clearly documented clinical indication as well as oral non-steroidal anti-inflammatory drugs, anti-diabetics, anti-hypertensives, and anti-psychotics were most often rated as having a higher probability of an adverse drug event, and should be prioritised for deprescribing in medication reviews in frail older adults.

## Introduction

The prevalence of multimorbidity (the presence of ≥ 2 medical conditions simultaneously) and polypharmacy (the use of ≥ 5 regular medications) continue to rise, particularly in older adults, i.e. those aged ≥ 65 years [1–3]. Older adults also experience age-related physiological changes that can impair their metabolism and excretion of medications, increasing sensitivity to their effects [4]. Furthermore, with advancing age, older adults are more likely to experience frailty, a distinctive health state related to the ageing process in which multiple body systems gradually lose their in-built reserves [5, 6]. These factors combine to make prescribing an increasingly more complex task in frail older adults, particularly in primary care for general practitioners (GPs), who are often tasked not only with balancing medications started for patients by several specialists, but also more frequently in initiating and managing medications for frail older adults without specialist input [7, 8].

The appropriateness of prescribing can be assessed using explicit criterion-based measures, one of which is the Screening Tool of Older Persons Prescriptions in Frail adults with limited life expectancy (STOPPFrail) [9]. STOPPFrail is a list of explicit deprescribing criteria for identifying potentially inappropriate medications (PIMs) in frail older adults with limited life expectancy [10, 11]. PIM prevalence is typically higher in frail older adults in nursing homes compared to their community-dwelling counterparts [12]. A recent study of two nursing homes in Ireland showed nearly all frail older adults were prescribed ≥ 1 STOPPFrail-defined PIM [13]. The prevalence of PIMs in older adults is rising and has been linked to an increased risk of adverse drug events (ADEs) and hospital admissions [14, 15]. Many ADEs associated with potentially inappropriate prescribing (PIP) are considered preventable, and up to 42% of serious, life-threatening, or fatal ADEs in primary care may be preventable [16]. PIP is also associated with significant avoidable expenditure in healthcare systems [17]. Furthermore, PIP has been shown to double the total direct healthcare costs compared to those without PIP [17]. Thus far, STOPPFrail’s use in a medication review intervention had been confined to just two research studies, both involving physicians applying the tool to frail older adults’ medications in hospital or transitioning from hospital to nursing homes [18, 19].

Cost avoidance has been defined as the reduction or elimination of additional expenditures that otherwise may have been incurred if an intervention had not occurred [20]. A cost avoidance study in an Irish hospital estimated a cost–benefit ratio of 8.64 due to ADEs prevented from pharmacist interventions over the course of a year [21]. Similarly, in the outpatient setting, the cost avoidance associated with clinical pharmacy interventions among patients with chronic disease produced a cost–benefit ratio of 5.98 [22]. A favourable return on investment of 1.71 has also recently been shown to be associated with pharmacists performing medication reviews during ward rounds in an internal medicine setting over one year [23]. Several studies have been conducted in nursing homes internationally to evaluate the cost savings associated with pharmacist-led interventions, but they have focused solely on attributing cost savings to deprescribed medications [24–26]. However, calls in the literature have been made to evaluate other contributions to the health economic value of pharmacist interventions, such as avoided hospital admissions [27]. To the research team’s knowledge, no study to date has evaluated the cost avoidance associated with pharmacist-led application of STOPPFrail in any setting.

### Aim

To evaluate cost avoidance associated with pharmacist-led application of STOPPFrail to frail older nursing home residents with limited life expectancy.

### Ethics approval

Ethical approval for this study was granted by the Clinical Research Ethics Committee of the Cork Teaching Hospitals (Reference: ECM 3 [ppp] Date: 16/11/2021).

## Method

### Setting

This descriptive cost avoidance study evaluated pharmacist-led application of STOPPFrail to frail older adults in six GP-affiliated nursing homes (5 private and 1 public) in Cork, Ireland from August 2021 to April 2023.

In Ireland, nursing homes may be classified as public or private or may be operated by charities or religious orders. Public nursing homes are run and funded by the Health Service Executive, Ireland’s public healthcare system. By contrast private nursing homes are run by private-sector agencies and provide approximately 75% of nursing home beds in Ireland [28, 29]. Within public and private nursing homes in Ireland medications are prescribed on-site by GPs who visit the homes (i.e. they are affiliated with ≥ 1 GPs), dispensed to nursing homes by community pharmacists who are located off-site, and administered by nurses during drug rounds. Multidisciplinary medication reviews are currently predominantly conducted by GPs in nursing homes with members of the nursing team and occasionally the dispensing community pharmacist at three-monthly intervals [30].

### Participants

Eligible participants were those aged ≥ 65 years residing in nursing homes, who met all of the following STOPPFrail inclusion criteria as decided by their GPs [10, 11]:

- End-stage irreversible pathology.
- Poor one-year survival prognosis.
- Severe functional and/or cognitive impairment.
- Symptom control is the priority as opposed to prevention of disease progression.

Potential participants were excluded from the study if they could not provide explicit informed consent (determined by patients’ GPs) to participate and there was no appropriate proxy to assent on their behalf to partake.

### Intervention

A research pharmacist (E.H.) with one year of community pharmacy experience reviewed patients' medical notes and medications and applied STOPPFrail (version 2) to them to identify PIMs for deprescribing [11]. Medications deemed clinically appropriate for potential deprescribing were flagged to participants’ GPs for review. The deprescribing recommendations were discussed in a meeting between the pharmacist and GP, and nursing staff where they were available to attend. Recommendations were recorded as accepted or not accepted by patients’ GPs. Patients were followed up with after six months to record changes to prescribed medications. Deprescribing recommendations found not to have been implemented or deprescribed PIMs that were re-started or increased back to their former dose were excluded from the cost avoidance estimates.

### Cost analysis

The cost-of-service provision was determined based on the cost of employing a pharmacist and GP. The mean time ± (standard deviation [SD]) for the pharmacist to conduct a review was 35 ± 9 min. This was calculated based on the time for the pharmacist to review patients’ medical records, create the deprescribing recommendations and communicate them to GPs in the multidisciplinary meeting. The hourly rate of employing a pharmacist was calculated from the mid-point of Ireland’s public healthcare system’s salary scale for a basic grade pharmacist: €59,417 [31]. The mean time ± (SD) per patient for GPs to consider the STOPPFrail recommendations with the pharmacist was 3 ± 1.2 min. This was calculated based on the time for GPs participating in the multidisciplinary meeting with the pharmacist to discuss and implement the deprescribing recommendations. The hourly rate of employing a GP was calculated based on the mid-point of the current Department of Health Northern Ireland salary scale for salaried GPs (£82,814); this was converted to euro using the conversion rate of 1.13 in June 2023 to €93,355.91 [32]. The Northern Ireland salary scale was used as there is no published scale for GPs in the Republic of Ireland. GP and pharmacist salaries were adjusted by factoring in employer-related costs and other overheads, based on guidance for conducting a budget impact analysis within Ireland’s healthcare system [33].

Cost avoidance was calculated based on the probability of an ADE (pADE) occurring if an accepted recommendation to deprescribe a STOPPFrail-defined PIM was not implemented by the GP, multiplied by the potential cost of an ADE, as per guidance on conducting cost avoidance studies [34]. Occasionally, PIMs had > 1 recommendation associated with them (e.g. folic acid prescribed for ≥ 4 months where there was also no documented indication - such as folate deficiency, malabsorption, methotrexate co-prescribed etc.). In this case, the more specific deprescribing recommendation was used to describe the PIM, i.e. folic acid prescribed for ≥ 4 months rather than the lack of a clinical indication. Each STOPPFrail-defined PIM and the associated recommendation(s) were provided in a Microsoft Word® document to a panel of four independent raters who had experience of providing care to patients in nursing homes (two GPs and two pharmacists). Where a PIM had ≥ 1 associated recommendation, the pADE occurring was only evaluated once per PIM. In addition, raters were provided with a list of patients’ comorbidities, age, gender, medications at baseline, and - where available/relevant - their laboratory parameters, blood pressures, weights, as well as other miscellaneous clinical information to help aid raters’ decision-making. A fictional sample case is provided in the Supplementary Material (Appendix 1). Each rater was asked to rate the pADE if the recommendation to deprescribe a PIM was not implemented, based on Nesbit et al. methodology [35]. The raters were provided with Table 1 of examples developed by the research team. Raters returned their independent ratings individually via email. To determine the final estimated pADE, a consensus approach was adopted, whereby 75% of the raters had to choose the same pADE score for consensus to be considered achieved, which is regarded as the median threshold to define consensus [36]. Two teleconference meetings were held with all four raters to establish consensus pADE ratings for PIMs which had failed to achieve consensus from the initial independent ratings. Prior to the meetings, raters were provided with the original ratings from other raters.

**Table 1:** Examples of STOPPFrail-defined PIMs and the associated pADE occurring if not deprescribed. *Source*: Nesbit et al. [35]

Probability of an adverse drug event (pADE) occurring	Probability [35]	Example
No harm expected	0	Centrum Advance® (multivitamin) continued for a patient for prophylaxis of hypovitaminosis in a patient with adequate nutrition
Very low	0.01	Cholecalciferol 800 units daily continued for a patient without vitamin D deficiency
Low	0.10	Atorvastatin continued for primary hypercholesterolemia which is currently well controlled with no prior major cardiovascular or cerebrovascular event
Medium	0.40	Tolterodine continued for a patient with persistent, irreversible urinary incontinence without painful detrusor overactivity
High	0.60	Olanzapine continued for a patient with dementia without current features of behavioural and psychiatric symptoms of dementia

While previous cost avoidance studies in Ireland [21, 37] have used the ADE cost determined by Rottenkolber et al. [38], this was an estimated ADE cost for patients in a hospital setting. Therefore, given the present study's nursing home setting, we used the ADE cost from Field et al. instead, which determined the cost of preventable ADEs amongst older adults in the ambulatory setting in the United States of America (USA) [39]. This estimate was $1,983 (95% confidence interval, $193–$3,773) and adjusted for inflation and purchasing power parity between Ireland and the USA to a final estimate of €2,270 for an ADE.

To calculate the total cost avoidance, the savings associated with deprescribed medications were added to the summed cost avoidance associated with preventing ADEs. Savings associated with deprescribed medications were calculated based on the publicly available drug reimbursement prices in Ireland for the least experience generic version of medications to provide the most conservative estimate [40], in addition to monthly pharmacy dispensing fees of €5.48 per item [41]. Following estimation of the cost of conducting the interventions and the resulting cost avoidance, the net cost benefit (Eq. 1) and cost–benefit ratio (Eq. 2) for providing the service over a 1-year period were calculated. The analysis was calculated from the perspective of the healthcare institution, as recommended by Irish budget impact analysis guidelines [33]. Discounting was excluded given the inclusion criterion of a poor one-year survival prognosis, i.e. all ADEs and costs were expected to occur within one year.

1 $$\text{Net cost benefit = Total cost avoidance} \left(\left[\text{pADE} \times \text{cADE}\right]+ \text{DCS} \right) \text{- cost of service (cGP + cPharm)}$$

Equation 1: Net cost benefit: pADE = probability of adverse drug event, cADE = cost of adverse drug event, DCS = Direct cost savings i.e. reimbursement cost of medications + €5.48 dispensing fee per item, cGP = cost of GP, cPharm = Cost of pharmacist.

2 $$\text{Cost benefit ratio} = \frac{\text{Net cost benefit}}{\text{Cost of service}}$$

Equation 2: Cost benefit ratio.

### Sensitivity analysis

As per Patanwala et al. [34], each parameter included in the net cost benefit equation should include a lower and upper range estimate to increase confidence in the results and transparency. Lower and upper limits for each parameter are shown in Table 2.

**Table 2:** Upper and lower limits of parameters in net cost benefit equation

Variable	Lower limit	Mean value	Upper limit	Rationale
Pharmacist time per patient	17 min	35 min	57 min	Minimum and maximum recorded time to review a patient in this study
GP time per patient	2 min	3 min	3.5 min	Minimum and maximum recorded time to review recommendations with pharmacist per patient in this study
Pharmacist salary (+ adjustments)	€45,150 (€60,975)	€59,417 (€80,243)	€80,303 (€108,449)	Lowest point on HSE basic grade pharmacist salary scale and highest point on senior grade pharmacist scale [31]
GP salary (+ adjustments)	€74,416 (€103,476)	€93,356 (€130,232)	€112,296 (€156,652)	Lowest and highest point of Department of Health Northern Ireland GP salary scale converted to euro [32]
pADE	Consensus probability score × 0.5	Consensus probability score	Consensus probability score × 2.0 capped at 1.0 total	As per Nesbit et al. methodology [35]
cADE	€221	€2,270	€4,321	95% CI estimates for cADE from Field et al. converted to euro and adjusted for inflation and PPP [39]

pADE = Probability of adverse drug event, cADE = Cost of adverse drug event, CI = confidence interval, HSE = Health Service Executive i.e. Ireland’s public healthcare system, PPP = Purchasing power parity

### Statistical analysis

All data were analysed using Microsoft® Excel.

### Reporting guidelines

This study was reported in accordance with the Consolidated Health Economic Evaluation Reporting Standards (CHEERS) 2022 statement [42].

## Results

Across the six nursing homes, 99 patients out of total population of 328 were recruited to the study. A total of 193 recommendations pertaining to 176 STOPPFrail-defined PIMs across 69 patients were reviewed by the rating panel. Nearly all deprescribed PIMs were categorised as having potential to cause an ADE (*n* = 170; 97%). Table 3 provides a breakdown of the pADE occurring for each type of STOPPFrail-defined PIM. Table 3 also shows that the modal rating for the pADE occurring if PIMs were not deprescribed was '*very low*' (43.7%), whilst 15.9% were rated as ‘*low*’, 32.4% as ‘*medium*’, and 4.5% as ‘*high*’. The most prevalent STOPPFrail-defined PIMs accepted for deprescribing were medications without a clearly documented indication (61/176; 34.7%), followed by vitamin D (32/176; 18.2%) and proton pump inhibitors being used at full therapeutic doses for a duration ≥ 8 weeks (16/176; 9.1%). The most frequent PIMs without a clearly document indication were: diuretics (11/61; 18%), proton pump inhibitors (9/61; 14.8%), oral antihistamines (6/61; 9.8%), other antidepressants e.g. mirtazapine/trazodone (5/61; 8.2%), and anti-emetics (4/61;6.6%). Calcium, vitamin D, folic acid, multivitamin preparations, and oral nutritional supplements all had a pADE rating of either 'very low' or 'no harm expected' from being deprescribed. When calcium, vitamin D, folic acid, multivitamin preparations, and oral nutritional supplements were excluded, the pADE occurring if PIMs were not deprescribed was 'very low' (18.1%), whilst 24.1% were rated as low, 49.1% as medium, and 6.9% as high.

**Table 3:** Consensus probability of an adverse drug event occurring by STOPPFrail potentially inappropriate medication type (*n* = 176)

STOPPFrail recommendation	No harm expected *n* (%)	Very low probability *n* (%)	Low probability *n* (%)	Medium probability *n* (%)	High probability *n* (%)	STOPPFrail recommendation *n* (% of total potentially inappropriate medications)
Medication with no clear indication	2 (33.3)	14 (18.1)	11 (51.4)	29 (49.1)	5 (62.5)	61 (34.7)
Vitamin D	0	32 (41.6)	0	0	0	32 (18.2)
Proton pump inhibitors	0	0	5 (14.3)	11 (19.3)	0	16 (9.1)
Folic acid	0	9 (11.7)	0	0	0	9 (5.1)
Oral nutritional supplements	0	8 (10.4)	0	0	0	8 (4.5)
Antihypertensives	0	0	0	6 (10.5)	1 (12.5)	7 (3.9)
Calcium supplements	0	7 (12.9)	0	0	0	7 (3.9)
Memantine	0	0	6 (17.1)	0	0	6 (3.4)
Antipsychotics	0	0	0	6 (10.5)	0	6 (3.4)
Medication for symptoms which have resolved	0	3 (3.9)	0	0	1 (12.5)	4 (2.3)
Lipid lowering medications	0	0	4 (11.4)	0	0	4 (2.3)
Multivitamin preparations	4 (66.6)	0	0	0	0	4 (2.3)
Anticholinergics or mirabegron	0	0	1 (2.9)	2 (3.5)	0	3 (1.7)
Medication that patient fails to take	0	3 (3.9)	0	0	0	3 (1.7)
Anti-diabetic medications	0	0	0	3 (5.3)	0	3 (1.7)
Anti-resorptive medications	0	1 (1.3)	1 (2.9)	0	0	2 (1.1)
NSAIDs	0	0	0	0	1 (12.5)	1 (0.6)
Total within each probability rating	6/176 (3.4%)	77/176 (43.7%)	28/176 (15.9%)	57/176 (32.4%)	8/176 (4.5%)	

A considerable cost avoidance for the base case of €61,336 was calculated over a 1-year period from the perspective of the healthcare institution. Cost avoidance per STOPPFrail-defined PIM that was accepted for deprescribing was €348.5 (€61,336/176 PIMs). Cost avoidance per patient was €619.6 (€61,336/99 patients). Cost of delivering the intervention was calculated as €2,589. A substantial net cost benefit of €85,909 and cost–benefit ratio of 33.2 was determined based on the cost avoidance and direct cost savings. The upper and lower range estimates for the cost avoidance, cost of delivering the intervention, net cost benefit, and cost–benefit ratio are shown in Table 4.

**Table 4:** Cost analysis of all pharmacist interventions (*n* = 176) over a one-year period

	**Base case** (lower–upper range estimates)
1. Cost avoidance	**€61,336** (€2,986–€225,729)
2. Cost of service Pharmacist wages GP wages	**€ 2,589** (€988–€5,320) **€2,292** (€822–€4,904) **€297** (€166–€416)
3. Direct cost savings	**€27,162**
4. Net cost benefit [(1 + 3) − 2]	**€85,909** (€24,827–€251,903)
5. Cost benefit ratio [4/2]	**33.2** (5.7–255.9)

In all scenarios examined as part of a one-way deterministic sensitivity analysis shown in Table 5, the cost–benefit ratio was positive. The greatest variance in the cost–benefit ratio (21.3–67.6) was seen when the combined GP and pharmacist time taken to conduct the reviews was varied. The greatest variance in the net cost benefit was seen when the ADE cost was varied (€30,545–141,327).

**Table 5:** Sensitivity analysis for cost–benefit ratios and net cost benefit

Variable	Lower limit (cost benefit ratio–net cost benefit)	Upper limit (cost benefit ratio–net cost benefit)
Pharmacist salary	Highest point on senior pharmacist pay scale (25.1–€87,502)	Lowest point on basic pharmacist pay scale (42.4–€83,297)
GP salary	Highest point on GP pay scale (32.4–€85,790)	Lowest point on GP pay scale (34.0–€86,040)
ADE cost	Lowest point of Field et al. 95% CI (11.8–€30,545)	Highest point of Field et al. 95% CI (54.6–€141,327)
ADE probability	− 50% probability score (21.3–€55,241)	+ 100% probability score (55.3–€143,159)
Time	Maximum combined time for GP/Pharmacist to conduct a review (21.3–€84,523)	Minimum combined time for GP/Pharmacist to conduct a review (67.6–€87,206)

ADE = Adverse Drug Event, GP = General Practitioner, CI = Confidence Interval

## Discussion

### Statement of key findings

This study has shown considerable cost avoidance from a pharmacist applying STOPPFrail to frail older adults in nursing homes through deprescribing and preventing potential ADEs. The cost–benefit ratio for the intervention was 33.2 and remains positive in all scenarios, even when lower and upper limit estimates were tested in one-way sensitivity analyses, and could potentially save up to €251,903 in the year following STOPPFrail application for this cohort of 69 patients. Most PIMs (97%) were determined to have the potential for an ADE to occur if the GP had not implemented the pharmacist’s deprescribing recommendation(s). PIMs without a clearly documented indication, oral non-steroidal anti-inflammatory drugs, anti-diabetics, anti-hypertensives, and anti-psychotics were most often rated as having a higher probability of an adverse drug event.

### Strengths and weaknesses

This is the first study to the authors’ knowledge to appraise cost savings associated with pharmacist-application of STOPPFrail. An independent multidisciplinary panel of raters was used to estimate the pADE ratings, who each had experience in providing care to nursing homes—therefore improving the validity of the pADE ratings, rather than the research team estimating pADE ratings which may be introduced bias into the study and overestimated cost avoidance. The study also included a real-world representative patient cohort who had high levels of multimorbidity and polypharmacy, and also included those with a dementia diagnosis with cognitive impairment. Despite their increasing presence in clinical practice, these patients are typically excluded—and therefore underrepresented—in research, meaning that this study may better reflect the cost savings that pharmacists can make in frail older adult cohorts [43].

A study limitation was the use of the Field et al. estimated ADE cost to calculate cost avoidance. Ideally, local data from Irish nursing homes would be used to inform the potential ADE cost. However, no such data yet exists, so imperfect data which best matched the patient cohort remained the only option. Given it is not possible to directly compare Ireland’s cost of nursing home care with that in the USA the use of the Field et al. estimate may reduce the confidence in this study’s findings, albeit the confidence intervals of the estimate were used in the sensitivity analyses which may attenuate this risk [44]. Furthermore, it may have enhanced the study to factor a rating of the potential ADE severity. While such rating taxonomies exist [16], they do not assign a cost to the potential severity of ADE chosen. Lastly, the cost-of-service provision for this study was based on operating costs, rather than implementation costs, the latter of which would be higher and may include costs like hardware, software, training, GP/nursing time to consent patients or patient proxies, and room rental costs. However, not all potential benefits were accounted for in this study either, which may counterbalance this, e.g. nursing staff time saved not administering medications that were deprescribed and GP time saved that would have been spent conducting medication reviews in the intervention’s absence.

### Interpretation

The significant cost–benefit ratio of 33.2 from this study is higher than what has previously been reported in other economic analyses of pharmacist-led interventions [21, 22, 37]. Gallagher et al. calculated the costs avoided as a result of pharmacist interventions in a hospital over one year and reported a cost–benefit ratio of 8.64 [21]. A similar study by Ronan et al. showed a cost–benefit ratio of 16.54 for a senior pharmacist and 23.99 for a basic grade pharmacist [37]. A study in Jordan found a cost benefit ratio of 5.98 of a clinical pharmacy intervention among hospital outpatients with chronic diseases [22]. None of the aforementioned studies [21, 22, 37] factored in the cost savings associated with deprescribed medications, which was included in the present study’s cost avoidance calculation [34]. There are several possible explanations for this study’s higher cost–benefit ratio. Firstly, this study utilised the Field et al*.* estimate ($1,983) [39] to calculate the ADE cost, whereas the other three studies utilised the Rottenkolber et al*.* estimate for costs associated with an ADE in hospitalised adults (€1,057). We acknowledge that this study’s cost avoidance calculation assumes patients will live long enough to experience an ADE, which may not be the case given their level of frailty. However, even if no ADEs occurred during the 12 months following the intervention, the direct cost savings arising from deprescribed medications alone would cover the cost of the intervention at least five-fold (Table 4).

This is the first study to have stratified STOPPFrail-defined PIMs according to the pADE occurring, which helps demonstrate PIMs that pose the greatest risk and which should be prioritised for deprescribing in frail older adults. Over one third of PIMs were rated as either ‘*medium*’ (32.4%) or ‘*high*’ (4.5%) for the pADE occurring, which is similar to the range of 2.0–66.4% reported in similar studies [21, 35, 37, 45, 46]. However, PIMs in this study were most commonly rated as having a ‘*very low*’ pADE occurring (43.7%), which is higher compared to these similar studies. While this may appear to reflect poorly on the potential clinical relevance of pharmacist recommendations, it is largely driven by vitamin D, calcium supplements, folic acid, and oral nutritional supplements which represented nearly a third of all PIMs (31.8%) and over three quarter of these were rated as having a ‘*very low*’ pADE occurring (76.6%). When recommendations pertaining to these four PIM categories were excluded, the percentage of PIMs rated as ‘*very low*’ pADE occurring decreased to 18.1%—with about half of the PIMs having a ‘*medium*’ pADE occurring (49.1%)—which may more accurately represent the overall clinical relevance of the intervention.

Of the PIMs that were rated as having a ‘*high*’ pADE occurring, nearly two thirds (62.5%) were for medications without a clear clinical indication. Documentation of indications for medications prescribed to nursing home residents has been highlighted as an issue by several studies [47–49]. Looking at other PIMs in this study, it should also be noted that all PIMs pertaining to anti-diabetics, anti-hypertensives, antipsychotics, and oral NSAIDs were deemed as having a ‘*medium*’ or ‘*high*’ pADE occurring (Table 3). Given the ADE probabilities for these four drug classes and medications without a clear clinical indication, (as well as the subsequent significant cost avoidance), it may be prudent to prioritise these medications for deprescribing during future medication-based interventions for frail older nursing home residents.

### Further research

Our findings show that pharmacist-led application of STOPPFrail to frail older nursing home residents has significant potential to reduce healthcare expenditure. However, given the limitations of a cost avoidance study based on a non-randomised, non-controlled prospective study, it would be useful in the future to perform a micro-costing analysis using real costs and outcomes in the context of a randomised controlled study [34]. As highlighted earlier, there is a paucity of research focusing on such cost avoidance in the nursing home setting. Therefore, it may be of value in future research to explore cost avoidance associated with pharmacist-led interventions in non-frail nursing homes residents at an earlier time point, not just those who are very frail with a limited life expectancy to maximise cost avoidance. Future research in this area of cost avoidance may be improved by the availability of accurate country-specific estimations of ADE cost in the nursing home setting. Lastly, in order to potentially increase the adoption of STOPPFrail by pharmacists providing medication reviews for nursing home residents; implementation science methodologies e.g. the Theoretical Domains Framework or Behavioural Change Wheel could be utilised to design a strategy to increase STOPPFrail’s utilisation [50].

## Conclusion

This study has robustly estimated that a pharmacist-led intervention using STOPPFrail in frail older adults in nursing homes results in considerable cost avoidance for the healthcare system - both indirectly due to avoided ADEs and directly due to deprescribed medications. The sensitivity analysis showed that the cost–benefit ratio for this intervention remained positive in all scenarios when lower and upper limit estimates for costs and benefits were evaluated. Therefore, these findings strengthen the case for wider implementation of such pharmacist interventions in frail older nursing home residents to reduce PIMs and patient harm, alongside substantial cost savings for healthcare systems.

## Supplementary Information

Below is the link to the electronic supplementary material.

Supplementary file1 (DOCX 23 kb)

## References

[1] Moriarty F, Hardy C, Bennett K, et al. Trends and interaction of polypharmacy and potentially inappropriate prescribing in primary care over 15 years in Ireland: a repeated cross-sectional study. BMJ Open. 2015;5: e008656. 26384726 10.1136/bmjopen-2015-008656 PMC4577876

[2] Whitty CJM, MacEwen C, Goddard A, et al. Rising to the challenge of multimorbidity. BMJ. 2020;368: l6964. 31907164 10.1136/bmj.l6964 PMC7190283

[3] Masnoon N, Shakib S, Kalisch-Ellett L, et al. What is polypharmacy? A systematic review of definitions. BMC Geriatr. 2017;17:230. 29017448 10.1186/s12877-017-0621-2 PMC5635569

[4] Mangoni AA, Jackson SHD. Age-related changes in pharmacokinetics and pharmacodynamics: basic principles and practical applications. Br J Clin Pharmacol. 2004;57:6–14. 14678335 10.1046/j.1365-2125.2003.02007.x PMC1884408

[5] Collard RM, Boter H, Schoevers RA, et al. Prevalence of frailty in community-dwelling older persons: a systematic review. J Am Geriatr Soc. 2012;60:1487–92. 22881367 10.1111/j.1532-5415.2012.04054.x

[6] Xue Q-L. The frailty syndrome: definition and natural history. Clin Geriatr Med. 2011;27:1–15. 21093718 10.1016/j.cger.2010.08.009 PMC3028599

[7] Baird B, Charles A, Honeyman M, et al. Understanding pressures in general practice [Internet]. London: The King’s Fund; 2016. p. 100. Report No.: 978 1 909029 61 3. www.kingsfund.org.uk/publications/pressures-ingeneral-practice. Accessed 23 Jun 2021.

[8] Damarell RA, Morgan DD, Tieman JJ. General practitioner strategies for managing patients with multimorbidity: a systematic review and thematic synthesis of qualitative research. BMC Fam Pract. 2020;21:131. 32611391 10.1186/s12875-020-01197-8 PMC7331183

[9] Thompson W, Lundby C, Graabaek T, et al. Tools for deprescribing in frail older persons and those with limited life expectancy: a systematic review. J Am Geriatr Soc. 2019;67:172–80. 30315745 10.1111/jgs.15616

[10] Lavan AH, Gallagher P, Parsons C, et al. STOPPFrail (screening tool of older persons prescriptions in frail adults with limited life expectancy): consensus validation. Age Ageing. 2017;46:600–7. 28119312 10.1093/ageing/afx005

[11] Curtin D, Gallagher P, O’Mahony D. Deprescribing in older people approaching end-of-life: development and validation of STOPPFrail version 2. Age Ageing. 2020;50:465–71. 10.1093/ageing/afaa159 32997135

[12] Lee Y, Jang S, Kang H-J, et al. Comparative analysis of potentially inappropriate medication use in long-term care facility residents and community-dwelling elders: a matched cohort study. Medicine (Baltimore). 2022;101: e31739. 36626501 10.1097/MD.0000000000031739 PMC9750672

[13] Heinrich CH, McCarthy S, McHugh S, et al. Identification of potentially inappropriate medications in frail older adults residing in long-term care: a retrospective chart review study. Drugs Real World Outcomes. 2023;10:97–106. 36436174 10.1007/s40801-022-00342-2 PMC9943820

[14] Pérez T, Moriarty F, Wallace E, et al. Prevalence of potentially inappropriate prescribing in older people in primary care and its association with hospital admission: longitudinal study. BMJ. 2018;363: k4524. 30429122 10.1136/bmj.k4524 PMC6233705

[15] Wallace E, McDowell R, Bennett K, et al. Impact of potentially inappropriate prescribing on adverse drug events, health related quality of life and emergency hospital attendance in older people attending general practice: a prospective cohort study. J Gerontol A Biol Sci Med Sci. 2017;72:271–7. 27466245 10.1093/gerona/glw140 PMC5233913

[16] Gurwitz JH, Field TS, Harrold LR, et al. Incidence and preventability of adverse drug events among older persons in the ambulatory setting. JAMA. 2003;289:1107–16. 12622580 10.1001/jama.289.9.1107

[17] Robinson EG, Hedna K, Hakkarainen KM, et al. Healthcare costs of adverse drug reactions and potentially inappropriate prescribing in older adults: a population-based study. BMJ Open. 2022;12: e062589. 36153031 10.1136/bmjopen-2022-062589 PMC9511550

[18] Curtin D, Jennings E, Daunt R, et al. Deprescribing in older people approaching end of life: a randomized controlled trial using STOPPFrail criteria. J Am Geriatr Soc. 2020;68:762–9. 31868920 10.1111/jgs.16278

[19] Loo CW, Ma BH-M. Reduction of polypharmacy in frail older patients in Hong Kong using STOPPFrail criteria. J Am Med Dir Assoc. 2023;24:1261–2. 37356809 10.1016/j.jamda.2023.05.019

[20] Campbell AR, Nelson LA, Elliott E, et al. Analysis of cost avoidance from pharmacy students’ clinical interventions at a psychiatric hospital. Am J Pharm Educ. 2011;75:8. 21451760 10.5688/ajpe7518 PMC3049667

[21] Gallagher J, Byrne S, Woods N, et al. Cost-outcome description of clinical pharmacist interventions in a university teaching hospital. BMC Health Serv Res. 2014;14:177. 24742158 10.1186/1472-6963-14-177 PMC4020601

[22] Al-Qudah RA, Al-Badriyeh D, Al-Ali FM, et al. Cost-benefit analysis of clinical pharmacist intervention in preventing adverse drug events in the general chronic diseases outpatients. J Eval Clin Pract. 2020;26:115–24. 31234234 10.1111/jep.13209

[23] Jermini M, Fonzo-Christe C, Blondon K, et al. Financial impact of medication reviews by clinical pharmacists to reduce in-hospital adverse drug events: a return-on-investment analysis. Int J Clin Pharm. 2024. 10.1007/s11096-023-01683-w. 38315303 10.1007/s11096-023-01683-w PMC10960916

[24] Frankenthal D, Lerman Y, Kalendaryev E, et al. Intervention with the screening tool of older persons potentially inappropriate prescriptions/screening tool to alert doctors to right treatment criteria in elderly residents of a chronic geriatric facility: a randomized clinical trial. J Am Geriatr Soc. 2014;62:1658–65. 25243680 10.1111/jgs.12993

[25] Chia H. Pharmacist review and its impact on Singapore nursing homes. Singapore Med J. 2015;59:493–501. 10.11622/smedj.2015133 PMC4582128 26451051

[26] Elliott RA, Thomson WA. Assessment of a nursing home medication review service provided by hospital-based clinical pharmacists. Aust J Hosp Pharm. 1999;29:255–60. 10.1002/jppr1999295255

[27] Deeks LS, Kosari S, Naunton M. Clinical pharmacists in general practice. Br J Gen Pract. 2018;68:320. 29954794 10.3399/bjgp18X697625 PMC6014419

[28] Daly M. European Social Policy Network Thematic Report on Challenges in long-term care. [Internet]. 2018. file:///Users/eoinhurley/Downloads/IE_ESPN_thematic%20report%20on%20LTC%20(1).pdf. Accessed 26 Feb 2024.

[29] Health Information and Quality Authority. Overview report on the regulation of designated centres for older persons—2019 [Internet]. Dublin; 2020. p. 51. https://www.hiqa.ie/sites/default/files/2020-12/DCOP_Overview_Report_2019.pdf. Accessed 26 Feb 2024.

[30] Health Information and Quality Authority. National Standards for Residential Care Settings for Older People in Ireland [Internet]. Dublin; 2016. p. 102. https://www.hiqa.ie/sites/default/files/2017-01/National-Standards-for-Older-People.pdf. Accessed 3 Apr 2023.

[31] Health Service Executive (HSE). Consolidated salary scales in accordance with the FEMPI acts, the public service agreements and the public service pay and pensions act 2017 [Internet]. 2023. https://www.hse.ie/eng/staff/resources/hr-circulars/final-1-march-2023-salary-scales.pdf. Accessed 3 Jul 2023.

[32] Department of Health Northern Ireland. Pay and conditions of service: remuneration of hospital medical and dental staff, doctors and dentists in public health, the community health service, and salaried dental staff. [Internet]. 2022. https://www.health-ni.gov.uk/sites/default/files/publications/health/doh-hsc-tc8-03-2022.PDF. Accessed 3 Jul 2023.

[33] HIQA. Guidelines for the Budget Impact Analysis of Health Technologies in Ireland 2018 | HIQA [Internet]. 2018. https://www.hiqa.ie/reports-and-publications/health-technology-assessment/guidelines-budget-impact-analysis-health. Accessed 30 Nov 2022.

[34] Patanwala AE, Narayan SW, Haas CE, et al. Proposed guidance on cost-avoidance studies in pharmacy practice. Am J Health-Syst Pharm. 2021;78:1559–67. 34007979 10.1093/ajhp/zxab211

[35] Nesbit TW, Shermock KM, Bobek MB, et al. Implementation and pharmacoeconomic analysis of a clinical staff pharmacist practice model. Am J Health-Syst Pharm. 2001;58:784–90. 11351918 10.1093/ajhp/58.9.784

[36] Diamond IR, Grant RC, Feldman BM, et al. Defining consensus: a systematic review recommends methodologic criteria for reporting of Delphi studies. J Clin Epidemiol. 2014;67:401–9. 24581294 10.1016/j.jclinepi.2013.12.002

[37] Ronan S, Shannon N, Cooke K, et al. The role of the clinical pharmacist in an Irish university teaching hospital: a mixed-methods study. Pharmacy. 2020;8. 10.3390/pharmacy8010014 PMC7151682 32019094

[38] Rottenkolber D, Hasford J, Stausberg J. Costs of adverse drug events in German hospitals–a microcosting study. Value Health J Int Soc Pharmacoeconomics Outcomes Res. 2012;15:868–75. 10.1016/j.jval.2012.05.007 22999137

[39] Field TS, Gilman BH, Subramanian S, et al. The costs associated with adverse drug events among older adults in the ambulatory setting. Med Care. 2005;43:1171–6. 16299427 10.1097/01.mlr.0000185690.10336.70

[40] Health Service Executive (HSE). Health Service Exectutive Primary Care Reimbursement Service. List of Reimbursable Items [Internet]. https://www.sspcrs.ie/druglist/pub. Accessed 15 Mar 2023.

[41] National Centre for Pharmacoeconomics. Guidelines for Inclusion of Drug Costs in Pharmacoeconomic Evaluations [Internet]. 12012022. https://www.ncpe.ie/wp-content/uploads/2022/01/Guidelines-for-Inclusion-of-Drug-Costs-in-Pharmacoeconomic-Evaluations-v3.1.pdf. Accessed 15 Mar 2023.

[42] Husereau D, Drummond M, Augustovski F, et al. Consolidated health economic evaluation reporting standards 2022 (CHEERS 2022) statement: updated reporting guidance for health economic evaluations. BMC Med. 2022;20:23. 35022047 10.1186/s12916-021-02204-0 PMC8753858

[43] Herrera AP, Snipes SA, King DW, et al. Disparate inclusion of older adults in clinical trials: priorities and opportunities for policy and practice change. Am J Public Health. 2010;100(Suppl 1):S105-112. 20147682 10.2105/AJPH.2009.162982 PMC2837461

[44] Department of Health. A value for money review of nursing home care costs [Internet]. 2021. https://www.gov.ie/pdf/?file=https://assets.gov.ie/207532/45745dcf-c907-4227-808e-667c4d4286b8.pdf#page=null. Accessed 26 Feb 2024.

[45] Kearney A, Walsh EK, Kirby A, et al. A budget impact analysis of a clinical medication review of patients in an Irish university teaching hospital. Glob Reg Health Technol Assess. 2018;5.

[46] Patanwala AE, Hays DP, Sanders AB, et al. Severity and probability of harm of medication errors intercepted by an emergency department pharmacist. Int J Pharm Pract. 2011;19:358–62. 21899616 10.1111/j.2042-7174.2011.00122.x

[47] Rojas-Fernandez CH, Goh J, Hartwick J, et al. Assessment of oral anticoagulant use in residents of long-term care homes: evidence for contemporary suboptimal use. Ann Pharmacother. 2017;51:1053–62. 28745065 10.1177/1060028017723348

[48] Bennett N, Malloy MJ, James R, et al. Prophylactic antimicrobial prescribing in Australian residential aged-care facilities: improvement is required. Drugs Real World Outcomes. 2022;9:561–7. 35962922 10.1007/s40801-022-00323-5 PMC9712891

[49] Kelleher JE, Weedle P, Donovan MD. The prevalence of and documented indications for antipsychotic prescribing in Irish nursing homes. Pharm J Pharm Educ Pract. 2021;9:160. 10.3390/pharmacy9040160 PMC8544697 34698248

[50] Atkins DL, West PR, Michie PS. The behaviour change wheel. London: Silverback Publishing; 2014.

