# Supplementary material for: Cost avoidance of pharmacist-led deprescribing using STOPPFrail for older adults in nursing homes
Source: Int J Clin Pharm. 2024 Jul 5;46(5):1163–71. doi: 10.1007/s11096-024-01749-3 (PMC11399282; doi:10.1007/s11096-024-01749-3)
Supplement: Supplementary file 1 — Supplementary file1 (DOCX 23 kb) [file 11096_2024_1749_MOESM1_ESM.docx]

**Supplementary Material**

**Appendix 1: Sample Case**

**Age:** 94 **Gender:** Male

| **Medical History**: | **Medications:** |
| --- | --- |
| 1. Atrial fibrillation 2. Hypertension 3. Heart failure 4. Hypercholesterolemia 5. Dementia 6. Type 2 diabetes mellitus 7. Chronic obstructive pulmonary disease | 1. Apixaban 5mg BD 2. Furosemide 40mg OD 3. Bisoprolol 10mg OD 4. Cholecalciferol 800 units OD 5. Simvastatin 10mg NOCTE 6. Folic acid 5mg OD 7. Fortisip^®^ compact protein (oral nutritional supplement) OD 8. Rivastigmine 9.5mg/24hour transdermal patch OD 9. Quetiapine 50mg TDS 10. Metformin 1,000mg BD 11. Tiotropium (Spiriva^®^) inhaler 18 micrograms: one inhalation OD |

**Laboratory Parameters: Other relevant information:**

- Most recent blood pressure 159/81 mmHg.
- Weight 73kg.
- Patient has not recently experienced any behavioural or psychiatric symptoms of dementia.
- Folic acid has been prescribed for the past 12 months.
- Malnutrition Universal Screening Tool (MUST) score: 0 – i.e. patient not currently at risk of malnutrition, so oral nutritional supplements not indicated.

| Folate (micrograms/L) | >20 |
| --- | --- |
| Total cholesterol (mmol/L) | 3.5 |
| LDL cholesterol (mmol/L) | 2.5 |
| HDL cholesterol (mmol/L) | 2.1 |
| eGFR (MDRD) ml/min/1.73m^2^ | 83.1 |
| HbA1c (mmol/mol) | 41 |

|  |  | **No risk** | **Very low risk** | **Low risk** | **Medium risk** | **High risk** |
| --- | --- | --- | --- | --- | --- | --- |
| Cholecalciferol | Lack of clear evidence to support the use of vitamin D to prevent falls and fractures, cardiovascular events or cancers. |  |  |  |  |  |
| Simvastatin | Lipid-lowering therapies (statins, ezetimibe, bile acid sequestrants, fibrates, nicotinic acid, lomitapide and acipimox). |  |  |  |  |  |
| Folic acid | Folic acid: Discontinue when treatment course is completed. The usual treatment duration is 1–4 months unless malabsorption,  malnutrition, or concomitant methotrexate use. |  |  |  |  |  |
| Fortisip® compact protein | Nutritional supplements: Discontinue when prescribed for prophylaxis rather than treatment of malnutrition. |  |  |  |  |  |
| Quetiapine | Neuroleptic antipsychotics in patients with dementia: Aim to reduce dose and discontinue these drugs in patients taking them for longer than 12 weeks if there are no current clinical features of behavioural and psychiatric symptoms of dementia (BPSD). |  |  |  |  |  |
| Metformin | De-intensify therapy. Avoid HbA1c targets (HbA1C <7.5% [58 mmol/mol] associated with net harm in this population). The goal of care is to minimise symptoms related to hyperglycaemia (e.g. excessive thirst, polyuria). |  |  |  |  |  |
